# Angiogenic properties of dehydrated human amnion/chorion allografts: therapeutic potential for soft tissue repair and regeneration

**DOI:** 10.1186/2045-824X-6-10

**Published:** 2014-05-01

**Authors:** Thomas J Koob, Jeremy J Lim, Michelle Massee, Nicole Zabek, Robert Rennert, Geoffrey Gurtner, William W Li

**Affiliations:** 1 MiMedx Group, Inc., 1775 West Oak Commons Ct., Marietta, GA, USA; 2 Division of Plastic and Reconstructive Surgery, Department of Surgery, Stanford University School of Medicine, Palo Alto, CA, USA; 3 The Angiogenesis Foundation, Cambridge, MA, USA

**Keywords:** Amnion, Chorion, Amnion/chorion grafts, dHACM, Angiogenesis, Growth factors, VEGF, Endothelial cells, Soft tissue regeneration, Wound healing, Chronic wounds

## Abstract

**Background:**

Chronic wounds are associated with a number of deficiencies in critical wound healing processes, including growth factor signaling and neovascularization. Human-derived placental tissues are rich in regenerative cytokines and have been shown in randomized clinical trials to be effective for healing chronic wounds. In this study, PURION® Processed (MiMedx Group, Marietta, GA) dehydrated human amnion/chorion membrane tissue allografts (dHACM, EpiFix®, MiMedx) were evaluated for properties to support wound angiogenesis.

**Methods:**

Angiogenic growth factors were identified in dHACM tissues using enzyme-linked immunosorbent assays (ELISAs), and the effects of dHACM extract on human microvascular endothelial cell (HMVEC) proliferation and production of angiogenic growth factors was determined *in vitro*. Chemotactic migration of human umbilical vein endothelial cells (HUVECs) toward pieces of dHACM tissue was determined using a standard *in vitro* transwell assay. Neovascularization of dHACM *in vivo* was determined utilizing a murine subcutaneous implant model.

**Results:**

Quantifiable levels of the angiogenic cytokines angiogenin, angiopoietin-2 (ANG-2), epidermal growth factor (EGF), basic fibroblast growth factor (bFGF), heparin binding epidermal growth factor (HB-EGF), hepatocyte growth factor (HGF), platelet derived growth factor BB (PDGF-BB), placental growth factor (PlGF), and vascular endothelial growth factor (VEGF) were measured in dHACM. Soluble cues promoted HMVEC proliferation *in vitro* and increased endogenous production of over 30 angiogenic factors by HMVECs, including granulocyte macrophage colony-stimulating factor (GM-CSF), angiogenin, transforming growth factor β3 (TGF-β3), and HB-EGF. 6.0 mm disks of dHACM tissue were also found to recruit migration of HUVECs *in vitro*. Moreover, subcutaneous dHACM implants displayed a steady increase in microvessels over a period of 4 weeks, indicative of a dynamic intra-implant neovascular process.

**Conclusions:**

Taken together, these results demonstrate that dHACM grafts: 1) contain angiogenic growth factors retaining biological activity; 2) promote amplification of angiogenic cues by inducing endothelial cell proliferation and migration and by upregulating production of endogenous angiogenic growth factors by endothelial cells; and 3) support the formation of blood vessels *in vivo*. dHACM grafts are a promising wound care therapy with the potential to promote revascularization and tissue healing within poorly vascularized, non-healing wounds.

## Background

Normal wound healing is a complex biological process requiring interactions among distinct resident cell types, as well as inflammatory cells, platelets, and stem cells. Growth of new blood vessels into the wound through angiogenesis is a critical aspect of this process, to promote the adequate delivery of nutrients and regulatory factors required for tissue remodeling and regeneration.

The term angiogenesis describes the growth of new blood vessels from preexisting ones. After acute tissue injury, there is a rapid cascade of events involving the local release of angiogenic cytokines, including vascular endothelial growth factor (VEGF), placental growth factor (PlGF), platelet-derived growth factor (PDGF), basic fibroblast growth factor (bFGF), transforming growth factor β (TGF-β), angiogenin, angiopoietin, and other factors [1,2] by damaged cells and platelets. These factors lead to rapid destabilization of local preexisting vessels and activation of local endothelial cells, a process amplified by further release of growth factors from monocytes, macrophages, and fibroblasts. The concentration gradient of angiogenic signals guides activated endothelial cells to proliferate, migrate, sprout, and invade the extracellular matrix (ECM) in coordinated fashion, whereby three dimensional remodeling of vascular tubes creates a functional new microcirculatory network. As part of this response, proteolytic enzymes, including matrix metalloproteinases (MMPs), are released by endothelial cells to disrupt interactions with neighboring cells, facilitating endothelial cell adhesion, migration, and growth factor signaling through the dynamic modulation of integrin receptor expression [2].

In chronic wounds, however, normal healing processes including angiogenesis are disrupted, resulting in delayed or inappropriate healing. Chronic wounds often occur in the presence of systemic diseases such as diabetes and atherosclerosis, which are accompanied by microvascular deficiencies [1]. Moreover, a number of cellular and molecular defects are associated with poor healing of chronic wounds, including growth factor signaling, imbalances of MMPs and tissue inhibitors of metalloproteinases (TIMPs), and impaired recruitment of progenitor cells, suggesting that the correction of these abnormalities may promote a more natural healing process [3,4].

Human amniotic membranes have been successfully used to treat chronic cutaneous wounds [5-8]. Allografts derived from human amniotic membrane exhibit low immunogenicity and have shown the ability to reduce inflammation, pain, and scarring, and accelerate wound healing [9-15]. In a recent clinical trial published in 2013, the healing rate of diabetic foot ulcers significantly increased following treatment with dHACM, compared to those treated with a standard therapeutic regimen, with 77% and 92% of dHACM wounds healed at weeks 4 and 6, respectively, compared to only 0% and 8% of controls (*p* ≤ 0.001) [13]. Treatment with dHACM allografts has also demonstrated improved healing in patients with a variety of additional wound types for which traditional therapies were ineffective, including venous leg ulcers, crush injury, arterial insufficiency, immunological skin disease/scleroderma, and snake bite [14]. Additionally, refractory wounds that healed after dHACM treatment were reported not to recur with long-term follow-up [15].

Beyond serving as a protective wound barrier, human amniotic membrane provides a biological matrix supporting cell proliferation and tissue ingrowth. Growth factors that play a role in normal wound healing have also been identified in both fresh and preserved amniotic tissues, including epidermal growth factor (EGF), bFGF, keratinocyte growth factor (KGF), TGF-α and -β, hepatocyte growth factor (HGF), and nerve growth factor (NGF) [16-18]. We thus hypothesized that one mechanism by which amniotic membrane therapy accelerates wound healing is through induction of angiogenesis.

To ensure that safely harvested amniotic tissue allografts preserve their bioactivity for clinical application, are stable for long-term storage, and are available for off the shelf use, MiMedx Group, Inc. (Marietta, GA) has developed a gentle cleansing and dehydration process (PURION® Process), described elsewhere [19-21]. PURION® Processed and dehydrated human amnion/chorion membrane (dHACM) allografts have recently been shown to contain a multitude of pro-angiogenic growth factors beyond those listed above, including PDGF-AA, PDGF-BB, PlGF, granulocyte colony-stimulating factor (GCSF), and VEGF, among others [22].

The goal of this study was to determine the ability of PURION® Processed and dehydrated human amnion/chorion membrane (dHACM) allografts to support and promote angiogenesis. A thorough characterization of the angiogenesis-related growth factors within EpiFix® dHACM advanced wound care product (MiMedx Group, Inc.) was performed. To examine the ability of dHACM to modulate endothelial cell behavior, human endothelial cell proliferation, migration, and their production of endogenous angiogenic factors *in vitro* in response to dHACM was determined. *In vivo* studies were further conducted to determine the ability of dHACM to support angiogenesis in a murine subcutaneous implantation model.

## Materials and methods

### Dehydrated human amnion/chorion membrane (dHACM)

dHACM is a dehydrated human allograft comprised of laminated amnion and chorion membranes derived from the placenta [19-21]. Human placentas were donated under informed consent following Cesarean sections, as regulated by the Food and Drug Administration’s (FDA) Good Tissue Practice and American Association of Tissue Banks (AATB). All donors were tested to be free of infectious diseases, including human immunodeficiency virus (HIV), human T-lymphotropic virus (HTLV), hepatitis B and C, syphilis, and cytomegalovirus (CMV). Amnion and chorion were isolated from placenta, processed with the proprietary PURION® Process that involves gentle cleansing of the layers, and then laminated to form the graft, which was dehydrated under controlled drying conditions [21]. A specific version of dHACM (EpiFix®, MiMedx Group) was used as the test material in this study; therefore, the results of this study apply only to PURION® Processed dehydrated human amnion/chorion composite grafts (dHACM).

### ELISA assays

The content of angiogenic growth factors in samples of processed, dehydrated human amnion/chorion grafts (dHACM) from eight donors was measured with standard enzyme-linked immunosorbent assays (ELISAs; RayBiotech, Inc., Norcross, GA). Weighed, minced dHACM samples were placed in lysis buffer containing protease inhibitors for 24 hours at 4°C. Samples were then homogenized, centrifuged to remove tissue residue, and the amount of specific angiogenic factors in the lysis buffer was measured in diluted aliquots with standard ELISAs. Growth factor content was normalized to the dry mass of starting tissue.

### *In vitro* proliferation of human microvascular endothelial cells

To prepare extracts of dHACM for cell culture experiments, sterilized grafts were minced and extracted in Medium 131 (Gibco, Life Technologies Corp. M-131-500, Carlsbad, CA), with and without supplement, at a concentration of 20 milligrams of tissue per milliliter of medium. After 24 hours of extraction at 4°C, the tissue residue was removed by centrifugation and the extract was sterile filtered. Previous studies have established that a significant amount of the growth factors and cytokines in dHACM elute from the tissue under these conditions [22].

Human microvascular endothelial cells (HMVECs; Gibco, Life Technologies Corp. C-011-5C) from adult dermis were plated on 96-well plates for 24 hours in Medium 131 with Microvascular Growth Supplement (Gibco, Life Technologies Corp. S-005-25). The Microvascular Growth Supplement was comprised of growth factors, hormones, and tissue extracts necessary for culture of HMVECs, and the final concentration of supplements in Medium 131 was 4.9% v/v fetal bovine serum, 1 μg/mL hydrocortisone, 3 ng/mL human fibroblast growth factor, 10 μg/mL heparin, 1 ng/mL human epidermal growth factor, and 0.08 mM dibutyryl cyclic AMP. After 24 hours to allow for cell adhesion, the medium was aspirated from the wells and replaced with one of the following: medium lacking supplement (negative control), medium plus supplement (positive control), or medium containing extracts of dHACM at 2 mg/mL, 1 mg/mL, or 0.5 mg/mL, both with and without supplement. After 72 hours, the plate was washed to remove unattached cells and a CyQuant assay (Molecular Probes, Life Technologies Corp. C7026) was performed to quantify DNA content (n = 5). DNA content was translated to cell number, using a standard curve of known cells as determined by counting on a hemocytometer.

### *In vitro* production of angiogenic growth factors by human microvascular endothelial cells

Human microvascular endothelial cells were cultured as described above in Medium 131 with Microvascular Growth Supplement for 24 hours on 96-well plates at a density of 3,500 cells/well. Following 24-hour culture to allow for cell adhesion, the cells were treated with dHACM extract in Medium 131 with supplement at 2, 1, and 0.5 mg/mL concentrations. After 72-hour treatment, the supernatant from 5 wells per sample group was recovered, pooled, and simultaneously tested for the presence of 60 growth factors, cytokines, and soluble growth factor receptors. Quantibody assays (Angiogenesis Array 1000, RayBiotech, Norcross, GA) were performed on the supernatant according to the manufacturer’s instructions and were quantified using a fluorescent microarray scanner (GenePix 4000B, Molecular Devices, Sunnyvale, CA). A CyQuant assay was subsequently performed, as described above, on the adherent cells to determine cell number.

### *In vitro* transwell migration studies with human umbilical vein endothelial cells

Using a modified Boyden chamber assay, human umbilical vein endothelial cells (HUVECs) were assayed for chemotactic cell migration toward dHACM tissue. Migration assays were performed in 24-well transwell inserts with 8 μm pore membrane filters (Corning, Corning, NY). 600 μL of supplement-free culture medium was loaded into the bottom wells, followed by the addition of differently sized portions of dHACM tissue, including 2.0 and 6.0 mm diameter disks (n = 4 dHACM tissue donors tested). Supplement-free Medium 200 (basal medium; Gibco, Life Technologies Corp. M-200-500) and Medium 200 containing Large Vessel Endothelial Supplement (complete medium; Gibco, Life Technologies Corp. A14608-01) acted as negative and positive controls, respectively (n = 4). The Large Vessel Endothelial Supplement contains fetal bovine serum, hydrocortisone, human epidermal growth factor, human basic fibroblast growth factor, heparin, and ascorbic acid, optimized for culture of HUVECs (Gibco, Life Technologies Corp. C-003-5C). 3.3 × 10^4^ HUVECs (passage 5) were seeded into the transwell inserts in 100 μl basal medium and cultured for 24 hours to permit migration.

After 24 hours, both sides of the inserts were rinsed with PBS, and non-migrating cells were removed with a cotton-tipped swab. Remaining cells were fixed in 4% formaldehyde in PBS for 30 minutes, and stained with 4’, 6-diamidino-2-phenylindole (DAPI) for 5 minutes prior to imaging in PBS on a fluorescence microscope (EVOS FL Auto, 10× objective, Life Technologies). Migrated cells were counted and averaged across four micrographs per insert.

### dHACM implantation and tissue harvest

Murine experiments conducted at Stanford University School of Medicine were approved by the Administrative Panel on Laboratory Animal Care (APLAC), protocol #20627. dHACM products from six donors were utilized in a simple subcutaneous implant model. Briefly, 4-month-old C57BL/6 J mice (Jackson Laboratories, Bar Harbor, ME) were anesthetized and prepped before a horizontal 6 mm incision was created on the dorsum. A subcutaneous pocket was bluntly dissected in the fascial plane underlying the panniculus carnosus, and a 5 × 5 mm square of dHACM was surgically placed. At days 3, 7, 14, and 28 post operation, a cohort of mice (n = 3 per time point) was sacrificed, and the implant and overlying skin were harvested for histological analysis. Uninjured skin and sham-implanted sites (injured skin) were also collected as references for normal and post-injury cutaneous vascularity.

### Histological analysis of implant neovascularization

Tissue was harvested and embedded in OCT (Sakura Finetek USA, Inc., Torrance, CA). 10 μm thick frozen sections were fixed in acetone and immunostained using an antibody against CD31 (1:200, Abcam, Cambridge, MA). Nuclei were stained with DAPI. Randomized, duplicate microvessel counts per high power field (400×) were conducted on each skin or intra-implant sample following CD31 staining. To avoid sampling error, microvessels were defined as distinct areas of positive staining having a length to width ratio greater than or equal to one, and in close association with nuclei.

### Statistical analyses

Statistical comparisons were performed by using two-tailed, unpaired Student’s t-tests with significance set at *p* ≤ 0.05 to compare treatment groups to their respective controls. Two sample, two-tailed power analyses (α = 0.05) demonstrated that the statistical power of all tests was greater than 0.89. All values were expressed as the mean ± standard deviation.

## Results

### Angiogenic factors in dHACM

ELISAs performed on dHACM samples showed quantifiable levels of the following angiogenic growth factors: angiogenin, angiopoietin-2 (ANG-2), epidermal growth factor (EGF), basic fibroblast growth factor (bFGF), heparin binding epidermal growth factor (HB-EGF), hepatocyte growth factor (HGF), platelet derived growth factor BB (PDGF-BB), placental growth factor (PlGF), and vascular endothelial growth factor (VEGF). The amount of each factor was normalized to the starting dry weight of the tissue (Table 1).

**Table 1:** Angiogenic growth factor and cytokine content in dHACM grafts (n = 8 donors)

	**Average content (pg/mg of dry tissue)**	**Standard deviation**
Angiogenin	89.410	10.504
Angiopoietin-2 (ANG-2)	13.065	10.063
EGF	6.018	3.852
bFGF	0.717	0.225
HB-EGF	0.651	0.064
HGF	245.418	103.302
PDGF-BB	96.109	29.057
PlGF	2.396	0.953
VEGF	5.117	4.588

### Endothelial cell proliferation

**Figure 1:**
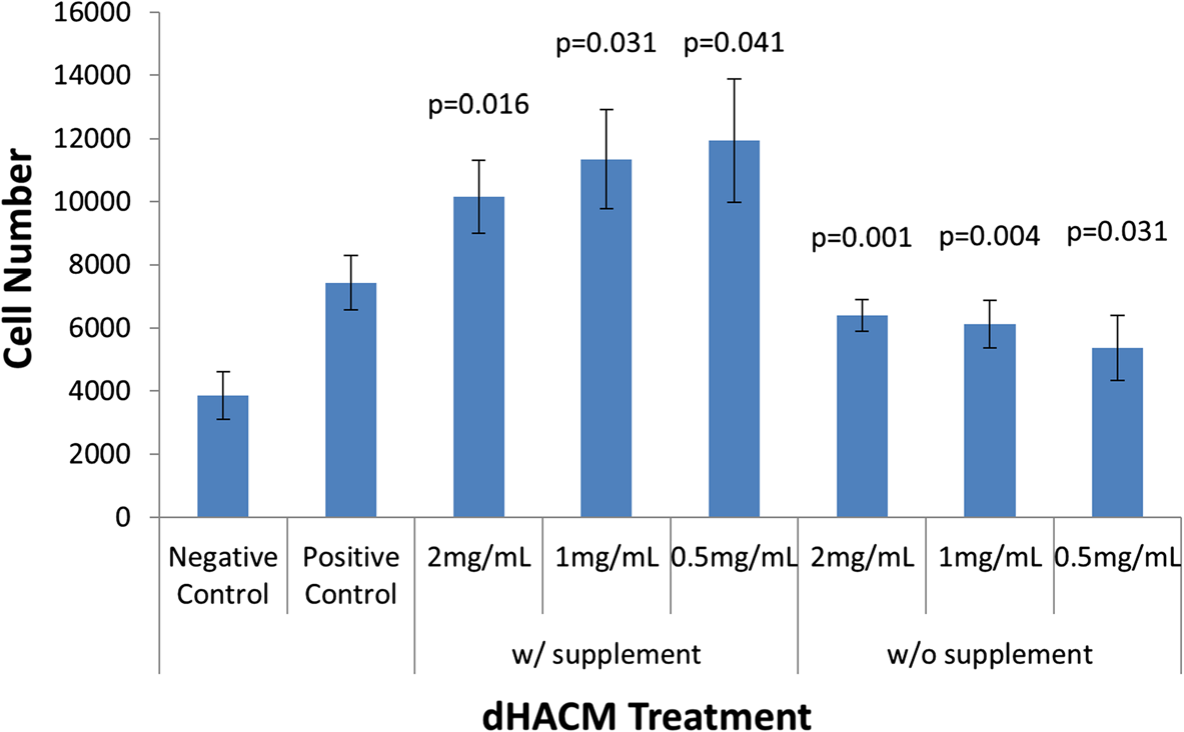
**Effects of extracts of dHACM on microvascular endothelial cell proliferation *in vitro. ***dHACM extracts promoted proliferation of HMVECs over controls; however, no dose response was observed at these concentrations. The p values shown indicate statistical significance from their respective controls. Values shown are means ± standard deviation (n = 5).

The human dermal microvascular endothelial cells proliferated to a minor extent in supplement-free medium (negative control), whereas inclusion of Microvascular Growth Supplement in the positive control wells caused the cells to nearly double in number (Figure 1). Extracts of dHACM caused proliferation of HMVECs in culture. At all concentrations of the extract, cells proliferated to a significantly greater extent than their respective controls (*p* ≤ 0.05) with or without supplement. There was no dose response observed among the three extract concentrations, indicating the maximum response was achieved with these concentrations.

### Growth factor production by microvascular endothelial cells

The effect of dHACM on growth factor production by human microvascular endothelial cells was examined by treating cells with extracts of dHACM for three days and measuring the amount of each growth factor in the culture medium at the end of the culture period. Since the extract caused endothelial cells to proliferate as shown in Figure 1, cell number was measured in each well after the culture medium was collected, and growth factor values were normalized on a per cell number. The amount of each growth factor in the extract itself was also measured and subtracted from that in the medium to calculate the amount of additional growth factor produced by the HMVECs themselves.

**Figure 2:**
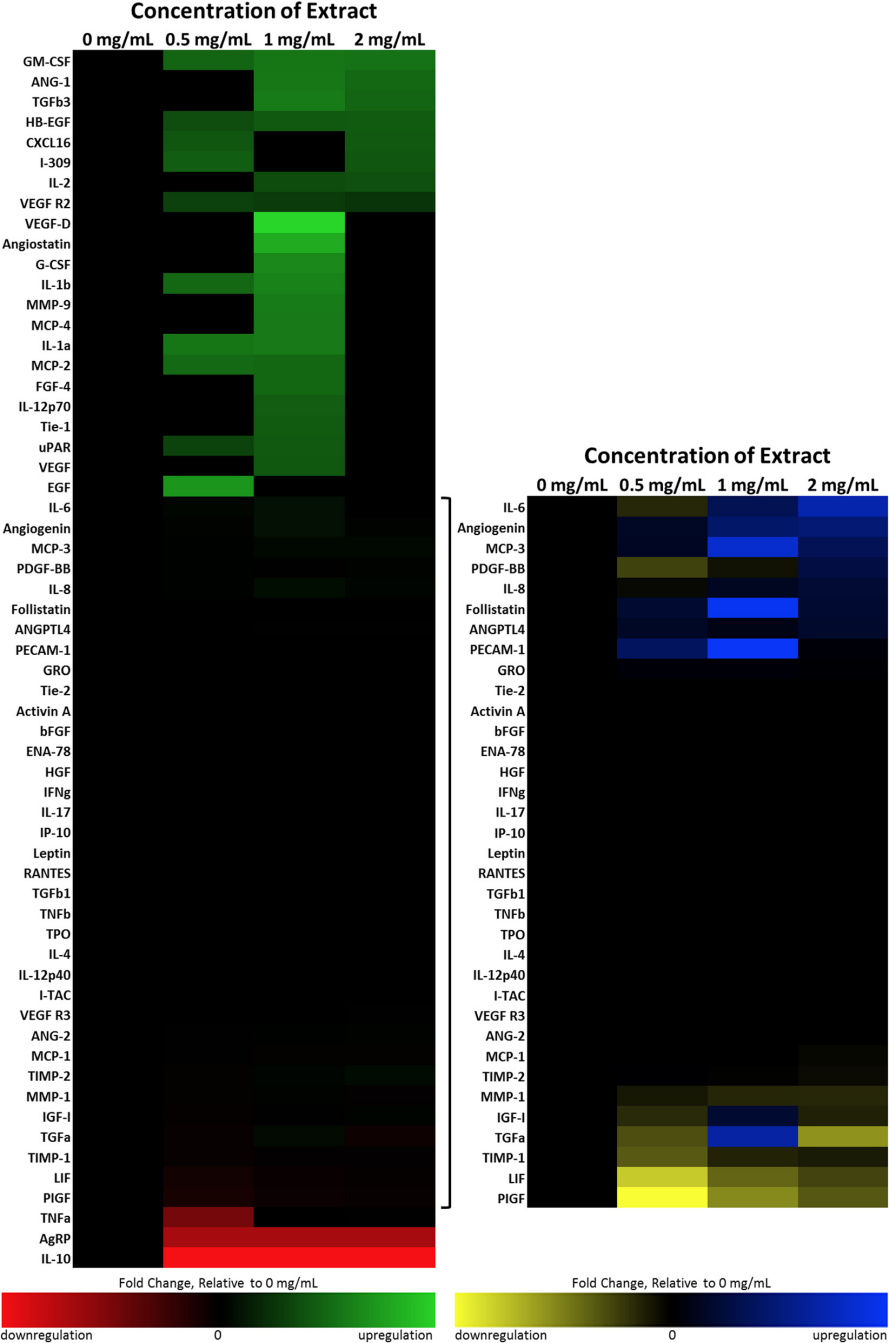
**Change in growth factor production by human microvascular endothelial cells when cultured in the presence of varying concentrations of dHACM extract.** Endothelial cells increased production of a number of angiogenic factors when cultured in the presence of dHACM extract, compared to untreated cells cultured without extract (green/blue). A few regulatory factors were also down regulated in the presence of extract (red/yellow), while many factors remained unchanged (black), relative to untreated controls.

The induction of specific growth factor production by endothelial cells in culture caused by extracts of dHACM is shown in Figure 2. HMVECs greatly increased production of a number of angiogenic factors including granulocyte macrophage colony-stimulating factor (GM-CSF), angiogenin, TGF-β3, HB-EGF, and interleukin-2 (IL-2) when cultured in the presence of dHACM extract, compared to untreated cells cultured without extract (Figure 2, green). HMVECs also increased production of molecules such as IL-6, angiogenin, and PDGF-BB to a lesser degree in the presence of extract (Figure 2, blue). A few regulatory factors including tumor necrosis factor α (TNF-α) and IL-10 were down regulated in the presence of extract (Figure 2, red/yellow), while many factors such as bFGF, HGF, and TGF-β1 remained unchanged (black), relative to untreated controls.

### Effects of dHACM on HUVEC migration *in vitro*

**Figure 3:**
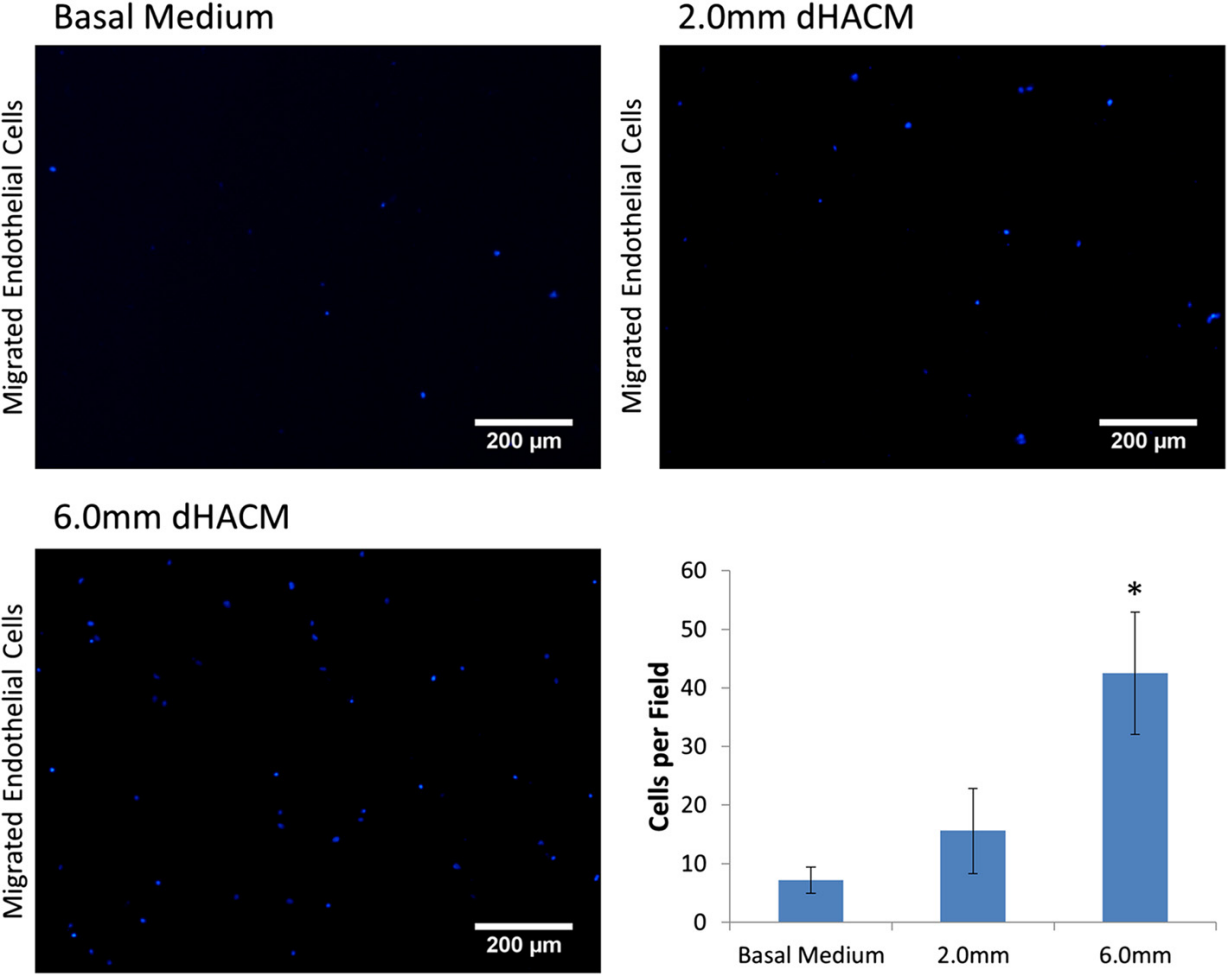
**Average number of migrated human umbilical vein endothelial cells (HUVECs) per field of view in response to dehydrated human amnion/chorion tissue allografts (dHACM).** Representative micrographs and cell counts indicated that greater migration was observed in response to larger samples, relative to their smaller counterparts. HUVEC migration in complete medium was significantly greater than all other samples (*p* ≤ 0.05). * indicates significantly greater migration than basal medium and 2.0 mm groups (*p* ≤ 0.05). Scale bar – 200 μm.

HUVEC migration in the basal medium and the smallest 2.0 mm samples was not significantly different; however, samples containing 6.0 mm diameter disks of dHACM tissue demonstrated significantly greater migration compared with basal medium and 2.0 mm samples (Figure 3). No experimental group reached the number of cells comparable to complete medium (260 ± 68 cells per field); however, the 6.0 mm diameter samples demonstrated that dHACM tissue in the culture medium was capable of directing endothelial cell migration *in vitro*.

### Neovascular response to dHACM following *in vivo* implantation

**Figure 4:**
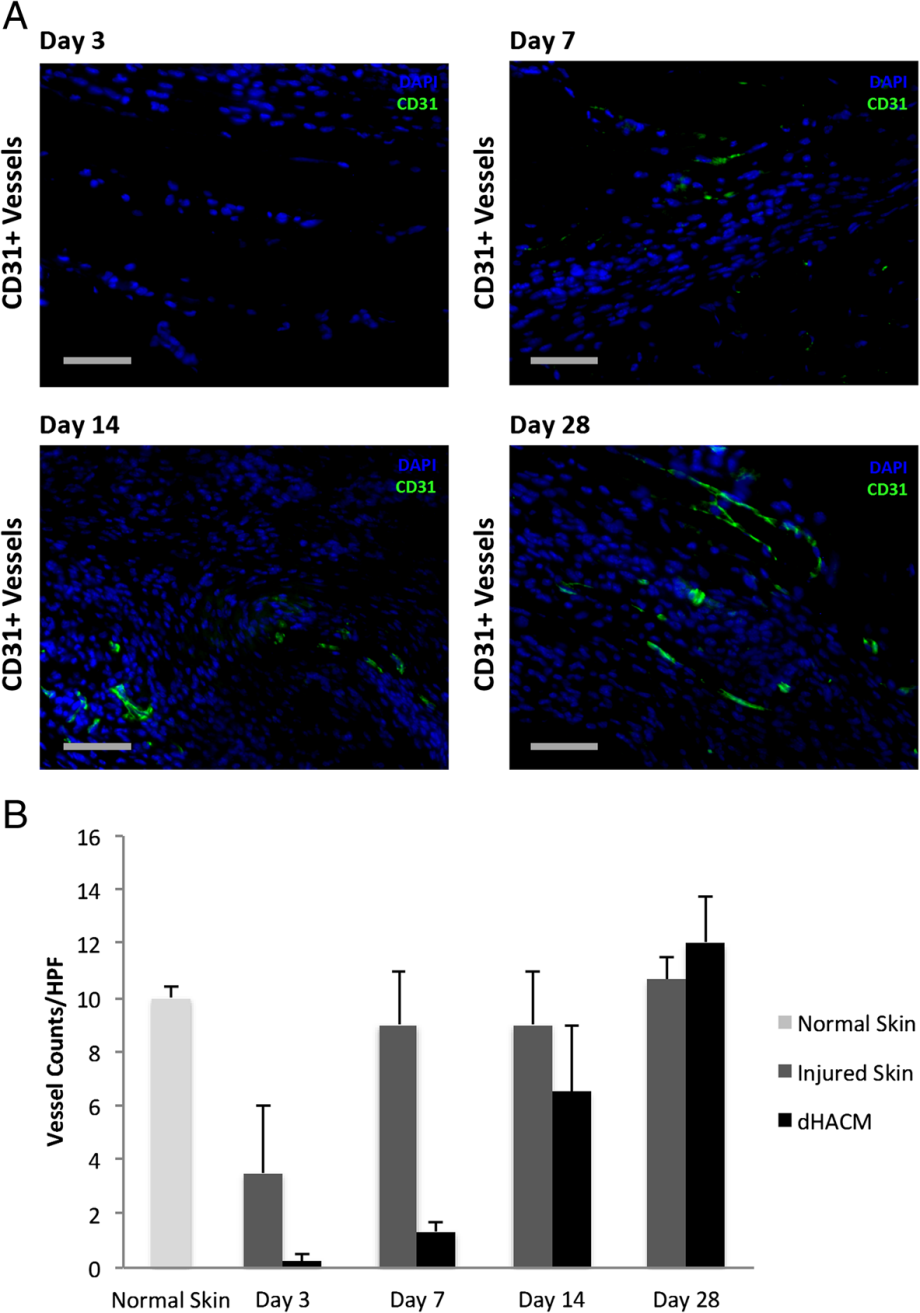
**Neovascular response following *in vivo *dHACM implantation.** An increasing number of CD31 positive microvessels were seen within the implanted dHACM over time, ultimately reaching the level of normal and previously injured skin by day 28. **(A)** CD31 positive microvessels in dHACM over 28 days were stained green, while cell nuclei were counterstained blue with DAPI. **(B)** Vessel counts demonstrated an increase in vascularization approaching that of healthy and healing skin after 28 days. Scale bar – 50 μm.

Immunohistochemical staining for the endothelial cell specific antigen CD31 was performed to characterize the neovascular response within implanted dHACM grafts themselves. The quantity of CD31 positive microvessels within the dHACM grafts were found to steadily increase through day 28 to levels similar to normal and healing skin, indicative of dynamic intra-implant neovascularization (Figure 4).

## Discussion

These studies clearly demonstrate that dehydrated human amnion/chorion membranes (dHACM) contain a large number of pro-angiogenic growth factors, including angiogenin, angiopoietin-2, EGF, bFGF, HB-EGF, HGF, PDGF-BB, PlGF, and VEGF. This partial list of growth factors does not encompass the entire array of physiologically important and biologically active molecules present in dHACM [22]. These particular growth factors, however, are likely to be responsible for the clinical benefits of this dHACM allograft, in relation to neovascularization and healing within chronic wounds. These soluble signals in dHACM also stimulated human microvascular endothelial cells to proliferate *in vitro*, and further, to increase production of a number of endogenous growth factors, cytokines, and receptors related to angiogenesis. Furthermore, dHACM tissue promoted chemotactic migration of human endothelial cells *in vitro*, suggesting that these soluble factors are capable of recruiting endothelial cells to promote wound re-vascularization. Our findings strongly support dHACM exerting therapeutic actions both directly and indirectly by activating multiple signaling pathways that promote angiogenesis within healing wounds.

Our previous work demonstrated that PURION® Processed dHACM tissue also retains a collection of growth factors, anti-inflammatory molecules, and tissue inhibitors of metalloproteinases that play other important roles in wound healing. Specifically, this material or its extracts, promoted the proliferation of dermal fibroblasts and the recruitment of progenitor cells *in vitro* and *in vivo *[22]. Combined with the present results, dHACM allografts contain a wide array of soluble signals, only a fraction of which have been identified to date, which may stimulate healing through a variety of signaling pathways and physiological mechanisms.

These multifunctional mechanisms offer advantages for dHACM as a treatment of difficult to heal chronic wounds. In normal wounds, the initial fibrin clot acts as both a matrix for repair and as a reservoir for growth and cell recruitment factors. Inflammatory cells, including neutrophils, monocytes, and lymphocytes then invade the wound and release further growth factors and cytokines that initiate wound repair mechanisms, including keratinocyte and fibroblast migration/proliferation, microvascular endothelial cell recruitment, proliferation, and angiogenesis, and nerve sprouting within the granulation tissue [23]. In chronic wounds, however, including diabetic and venous ulcers characterized by deficiencies in vascularization and cytokine signaling, the exogenous delivery of key cytokines and growth factors may be necessary to restore a molecular balance and achieve healing [24].

Single growth factors may be insufficient to overcome to multiple deficiencies in most chronic wounds, as demonstrated by the modest efficacy and limited utility of recombinant human PDGF (becaplermin) as a single agent as shown in a meta-analysis of well-designed clinical trials [25]. By contrast, dHACM contains additional angiogenic factors, specifically VEGF and bFGF (FGF2), both potent angiogenic cytokines that promote endothelial cell proliferation and migration [26]. We also identified PlGF which not only has direct angiogenic effects, but acts synergistically with VEGF to stimulate wound angiogenesis [27]. The growth factors angiopoietin-1 and 2 are critical for regulating the stabilization and remodeling of blood vessels [23,26]. Our finding that dHACM also stimulates human microvascular endothelial cells to increase production of a variety of angiogenic cytokines and growth factors suggests this material’s ability to amplify the initial signals provided by the allograft itself, potentially even beyond the lifespan of the allograft.

Previous studies of amniotic membranes in regulating angiogenesis have reported conflicting data, with some describing enhanced vascularization while others have observed inhibition of angiogenesis. Anti-angiogenic effects have been widely reported in cases of ocular/corneal surgery [28], while improved vascularization and healing have been reported when used to promote healing of cutaneous wounds [29,30]. This dichotomy may be explained in part by the presence of both anti-angiogenic (including thrombospondin-1, endostatin, and anti-angiogenic TIMPs) [31] and pro-angiogenic factors (including VEGF and PDGF) [32,33] in amniotic tissues. Combined with the previously described role of the local environment in regulating angiogenesis, the specific response to the amniotic membrane graft cytokine milieu may thus be largely dependent on the target tissue and the location of the implantation.

In this study, subcutaneous implantation of dHACM tissues in a murine model demonstrated a steady increase in vascularization through day 28. More specifically, dHACM implants went from being completely avascular following implantation, to having a vascular density similar to both normal and healed skin within 4 weeks. This dynamic vascular remodeling is in line with our *in vitro* findings, and consistent with the time course of clinical trial data [13], supporting the retention of biological activity by growth factors present in PURION® Processed dHACM grafts. The prolonged pro-angiogenic effects from a single implant may offer potential practical benefits compared to advanced wound interventions that require more frequent (daily or biweekly) applications.

The angiogenic properties of amniotic membrane grafts demonstrated here apply only to PURION® Processed, dehydrated amnion/chorion laminated grafts produced by MiMedx. The PURION® Process has been optimized to preserve the bioactivity of native amniotic growth factors and cytokines, while other amniotic membrane grafts processed by different methods may not retain biologically active angiogenic factors and, therefore, may lack the ability to stimulate angiogenesis. This is particularly true for single layer amnion products, which are inherently much thinner and therefore contain far fewer growth factors, as well as decellularized amniotic membrane grafts, which have been processed to remove cells and soluble factors from the tissue matrix.

The clinical value of dHACM grafts for use as therapy in non-healing wounds has been demonstrated by clinical research, even as further translational studies are underway. Treatment with dHACM allografts was reported to improve healing in patients with a variety of wound types for which traditional therapies were ineffective [14]. Additionally, refractory wounds that healed after dHACM treatment were reported not to recur with long-term follow-up [15]. Finally, in a small, prospective, randomized clinical trial, Zelen et al. demonstrated a significant increase in the healing rate of diabetic foot ulcers treated with dHACM compared to those treated with a standard therapeutic regimen, with 77% and 92% of dHACM wounds healed at weeks 4 and 6, respectively, compared to only 0% and 8% of controls [13]. The aggregate data of dHACM suggests this material could be studied for comparative effectiveness with other available wound healing products.

## Conclusions

In summary, dHACM allografts derived from human placenta contain multiple angiogenic growth factors with retained biological activity, directly stimulate angiogenesis, and encourage amplification of angiogenic signaling cues by inducing production of endogenous growth factors from human endothelial cells *in vitro*. The angiogenic effects are prolonged to at least 28 days. These properties help explain the effectiveness of dHACM reported in clinical studies, and suggest the potential of dHACM grafts to promote revascularization and healing within poorly vascularized, non-healing wounds and other tissues.

## Abbreviations

AATB: American Association of Tissue Banks; ANG-2: Angiopoietin-2; APLAC: Administrative Panel on Laboratory Animal Care; bFGF/FGF2: Basic fibroblast growth factor; CMV: Cytomegalovirus; DAPI: 4’, 6-diamidino-2-phenylindole; dHACM: Dehydrated human amnion/chorion membrane; ECM: Extracellular matrix; EGF: Epidermal growth factor; ELISA: Enzyme-linked immunosorbent assays; FDA: Food and Drug Administration; GCSF: Granulocyte colony-stimulating factor; GM-CSF: Granulocyte macrophage colony-stimulating factor; HB-EGF: Heparin binding epidermal growth factor; HGF: Hepatocyte growth factor; HIV: Human immunodeficiency virus; HMVEC: Human microvascular endothelial cell; HTLV: Human T-lymphotropic virus; HUVEC: Human umbilical vein endothelial cell; IL: Interleukin; KGF: Keratinocyte growth factor; MMP: Matrix metalloproteinase; NGF: Nerve growth factor; PDGF: Platelet derived growth factor; PlGF: Placental growth factor; TGF: Transforming growth factor; TIMP: Tissue inhibitor of metalloproteinases; TNF: Tumor necrosis factor; VEGF: Vascular endothelial growth factor; VEGF-R2: VEGF receptor 2.

## Competing interests

This work was funded by MiMedx Group, Inc. TJK, JJL, MM, and NZ are employees of MiMedx. WWL is a consultant to MiMedx. RR and GG report no conflicts of interest.

## Authors’ contributions

TJK participated in the design of the studies, interpretation of data, and drafted the manuscript. JJL carried out the *in vitro* migration studies and helped draft the manuscript. MM carried out the proliferation studies and performed the ELISAs. NZ carried out the growth factor production studies and performed the ELISAs. RR carried out the *in vivo* neovascularization studies. GG participated in design of the *in vivo* neovascularization studies. WWL participated in interpretation of the data and revision of the manuscript. All authors read and approved the final manuscript.
